# Reperfusion of occluded branch retinal arteries by transluminal Nd:YAG laser embolysis combined with intravenous thrombolysis of urokinase

**DOI:** 10.1042/BSR20170930

**Published:** 2018-01-19

**Authors:** Fang Chai, Shanshuang Du, Xiquan Zhao, Runsheng Wang

**Affiliations:** Shanxi Ophthalmic Medical Center, Xi’an No. 4 Hospital, Affiliated Guangren Hospital, School of Medicine, Xi’an Jiaotong University, Xi’an 710004, China

**Keywords:** Branch retinal artery occlusion, Emboli, Nd:YAG laser, Urokinase

## Abstract

Purpose: To report successful treatment with transluminal Nd:YAG laser embolysis (TYE) combined with urokinase thrombolysis for reperfusion of occluded branch retinal arteries with visible emboli.

Methods: A total of 34 eyes from 34 patients with acute, severe vision loss secondary to a branch retinal artery occlusion with visible emboli and retinal whitening were examined. Each patient was administered TYE therapy, which focused on the embolus, using an ocular contact lens; a 0.3–0.9 mJ laser pulse was delivered directly and gradually according to the reaction. Fundus photographs and fundus fluorescein angiography (FFA) were obtained before and immediately after the laser treatment. All patients received urokinase thrombolysis therapy drops intravenously for 5 days at 10–20 u/d. The follow-up period ranged from 6 to 14 months after therapy. The morphological characteristics of FFA associated with obstruction recovery of arterial fluorescence filling and visual function were analyzed.

Results: After TYE therapy, FFA examinations showed that the retinal artery and its branches exhibited completely restored blood flow without obstruction in 13 eyes, accounting for 38.2% of the cases. The blood flow was mostly recovered in 11 eyes (32.4% of patients). FFA examinations following the combined intravenous urokinase thrombolysis therapy showed that the retinal artery and its branches exhibited completely restored blood flow after obstruction in 16 eyes (47.1% of patients). The blood flow was mostly recovered in 15 eyes (44.1% of patients).

Conclusion: TYE combined with urokinase thrombolysis is effective for reperfusion of occluded branch retinal arteries and improving visual recovery in patients with visible emboli.

## Introduction

Central retinal artery occlusion (CRAO) and branch retinal artery occlusion (BRAO) result in partial or complete retinal ischemia and sudden loss of vision. In most instances, these conditions are thought to be caused by an embolus, intraluminal thrombus, or atherosclerotic plaque. These conditions most commonly occur in older adults with a history of systemic hypertension, diabetes mellitus, carotid atherosclerosis, or cardiac valvular disease. Patients typically present with an abrupt, painless decrease in vision to the finger counting or light perception level. Acutely, the eye might demonstrate an afferent pupillary defect, slow development of a cherry-red spot in the macula with opacification of the surrounding retina, “boxcarring” of the blood column in the retinal vessels, or intra-arterial emboli. No effective treatment regimen has been developed [1,2]. Transluminal Nd:YAG laser embolysis (TYE) represents a therapeutic approach used for retinal vascular occlusive diseases. The main indication is BRAO with a visible embolus; this technique is not applicable for CRAO. This method consists of disrupting the intravascular embolus using a 1064 nm Nd:YAG laser focused on the embolus surface. Recent studies have indicated that Nd:YAG laser embolysis results in successful restoration of retinal blood flow and improves the best-corrected visual acuity (BCVA). The embolus is disintegrated by the laser, and the blood flow is partially restored; however, the embolus or emboli and debris remain in the vasculature [3–5], requiring further thrombolytic therapy.

To improve the therapeutic effect, we developed a novel method of TYE combined with urokinase thrombolysis therapy to reperfuse occluded branch retinal arteries with visible emboli. We sought to prospectively evaluate this method for safety and efficacy for treating patients with severe vision loss from BRAO with a visible embolus. In the present study, we evaluated the clinical effect of TYE combined with urokinase thrombolysis therapy for branch artery occlusion.

## Methods

This retrospective study was approved by the Institutional Review Board of Shanxi Ophthalmic Medical Center, Xi’an No. 4 Hospital. Patients were recruited from the same academic department (Ophthalmology Ward V & VI) in our hospital. Informed consent was obtained from all individual participants included in the study. The TYE treatments were performed by the same surgeon (Dr Wang Runsheng) to minimize bias due to different procedures and levels of experience.

### Patient selection

The present study included patients with BRAO or hemiretinal arterial occlusion who had visited Xi’an No. 4 Hospital between August, 2010 and April, 2015 due to vision loss or a defect of the visual field. Patients with opaque media, fundus photography, or fundus fluorescein angiography (FFA) examinations of the blocked artery that indicated invisible emboli, other eye diseases that affected vision or urokinase application contraindications were excluded. In addition, patients with a follow-up of less than 6 months (*n*=6) were excluded. In total, 34 eyes from 34 patients with acute BRAO were included in the analyses.

### Ophthalmic examinations

All patients underwent a complete ophthalmic examination that included measurement of the BCVA, slit-lamp biomicroscopy, indirect ophthalmoscopy, fundus photography (VX-10; Kowa OptiMed, Tokyo, Japan), FFA, and optical coherence tomography (OCT) imaging.

### Surgical procedures

A Goldmann three mirror lens was used to focus an Nd:YAG laser on the arterial embolus. The laser pulse was delivered starting at the lowest power possible (0.3 mJ) and increased to 0.9 mJ, until the embolus was partially or completely disrupted. After the embolus was dissolved, a small amount of vitreous hemorrhage was noted inferior to the optic disc. Intravenous urokinase thrombolysis was performed the next day after TYE in all the patients. Then, 10–20 million units of urokinase was added to 250 ml of 0.9% sodium chloride solution and administered in an intravenous drip for 30–40 min twice per day for five consecutive days. The follow-up examinations were performed on post procedure day 1, and the subsequent follow-up depended on the clinical course. FFA was obtained at the time of diagnosis and post-treatment.

### Statistical analysis

The demographic details, complications, obstruction site, and preoperative and postoperative data were compared between the TYE group and TYE combined intravenous urokinase thrombolysis therapy group. And the visual acuity was converted into the LogMAR value for the statistical analyses. GraphPad Prizm 5 software (GraphPad Software Inc., San Diego, CA, U.S.A.) and SPSS version 22.0 for windows (SPSS Science Inc., Chicago, IL, U.S.A.) statistical software packages were used. A *t*-test analysis was used for data obtained from TYE combined intravenous urokinase thrombolysis therapy group and peroperative patients. The final visual acuity between the two treatment groups was compared using Fisher’s exact test. For all tests, two-sided *P* values of 0.05 were considered statistically significant.

### Ethical approval

All procedures performed in the studies involving human participants were in accordance with the ethical standards of the institutional and/or national research committee and with the 1964 Helsinki declaration and its later amendments or comparable ethical standards. For this type of study, formal consent was not required.

## Results

**Figure 1:**
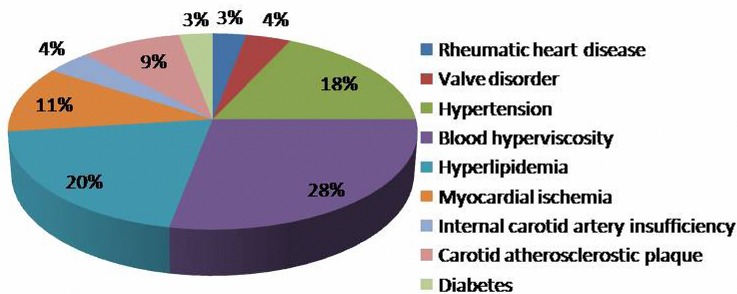
Combined diagnoses proportion chart

**Figure 2:**
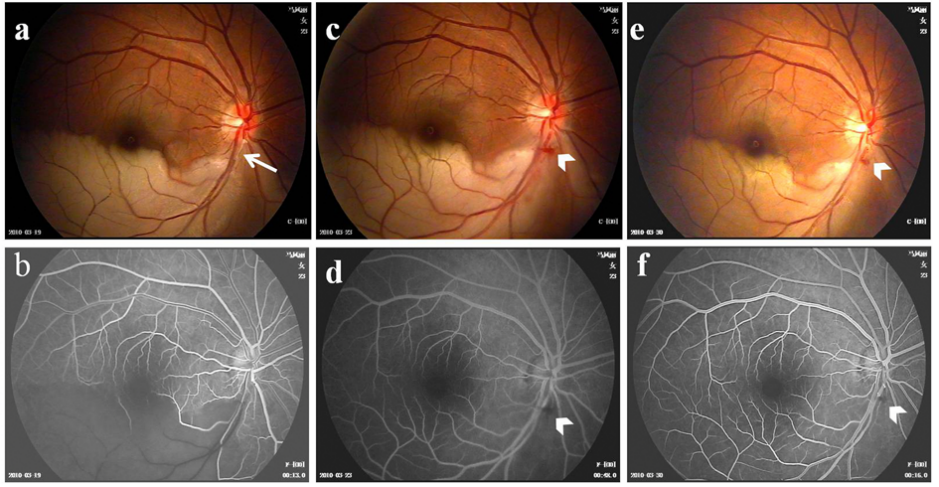
Color fundus images and fluorescein angiography (FFA) showing branch retinal artery obstruction (**a**) Color fundus photograph demonstrating an intraluminal embolus (arrow) and whitening of the retina along the inferior and temporal vascular arcades. (**b**) FFA of the same patient showing obstructed blood flow at the site of the embolus and a downstream filling defect. (**c**) Color fundus image of the same patient taken one day after Nd:YAG laser embolysis (TYE). Note the small amount of preretinal hemorrhaging (arrowhead) around the disc. (**d**) FFA of the same patient one day after TYE showing perfusion at the site of the embolus and restoration of the downstream blood flow. (**e**) Color fundus image taken 5 days after TYE combined with urokinase intravenous thrombolysis. (**f**) FFA at 5 days after TYE combined with urokinase intravenous thrombolysis showing a patent retinal artery with good blood flow and no neovascularization.

**Figure 3:**
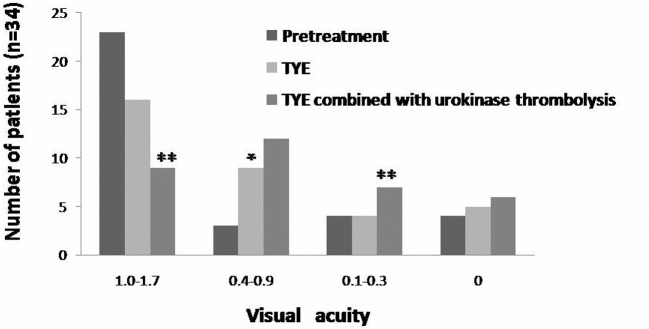
Visual acuity distribution The distribution of preoperative BCVA and postoperative BCVA following TYE combined with intravenous urokinase thrombolysis. Fisher’s exact test, and the ** indicates a *P*<0.05; Student’s *t*-test, and the * indicates a *P*<0.05.

**Figure 4:**
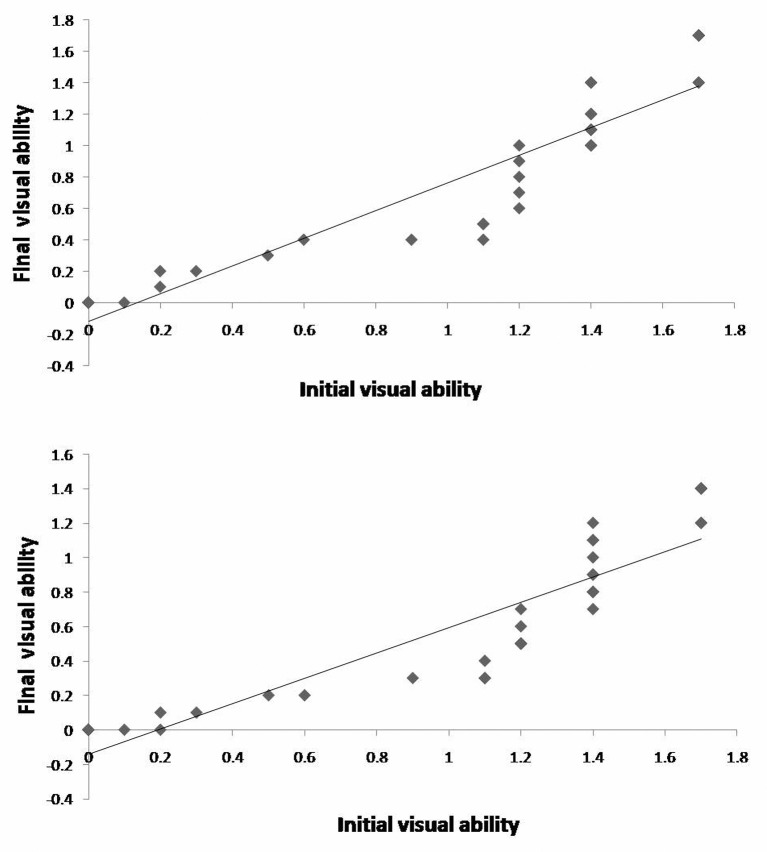
Preoperative best-corrected visual acuity (BCVA) and postoperative BCVA following TYE combined with intravenous urokinase thrombolysis Scattergram showing the relationship between the initial visual acuity and the final visual outcome after transluminal Nd:YAG laser embolysis (top) combined with intravenous urokinase thrombolysis (bottom).

Most of the patients had comorbid with high blood pressure, coronary heart disease, and other past medical history (Figure 1 and Table 1). Fundus examinations revealed intraretinal edema in the inferior macula with retinal whitening, in addition to a visible Hollenhorst plaque embolus in the associated branch of the retinal artery (Figure 2a). FFA revealed decreased arterial flow distal to the embolus and foveal ischemia (Figure 2b). For all of the patients on thrombolytics, Nd:YAG laser treatment resulted in retinal artery embolus rupture or disruption, immediate thickening of the artery diameter at the obstructed area (Figure 2c), and an alight reddening of the retinal occlusion. During the laser treatment, retinal hemorrhage occurred in two eyes, and vitreous hemorrhage was found in one eye. When combined with the urokinase intravenous infusion, the retinal occlusion area gradually became rosy (Figure 2e). No retinal vitreous hemorrhage and systemic complications were found during thrombolytic therapy. Based on the dynamic contrast cycle of fluorescence filling during early recovery, the results were divided into the following groups: complete fluorescein filling of the artery and its branch vessels was defined as complete recovery of the obstruction; 2/3 fluorescein filling of the branch vessels was defined as mostly restored; 1/3 fluorescein filling of the branch vessels was defined as partially restored; and the absence of dye filling in any of branch vessels was defined as ineffective. The changes in the best-corrected visual acuity (BCVA) values before and after the procedure are shown in Figures 3 and 4. All patients were observed after TYE combined with intravenous urokinase thrombolysis for a minimum of 6 months (range 6–14 months). No patients developed optic nerve dysfunction or retinal detachment. No instances of retinal neovascularization or choroidal neovascularization occurred during the follow-up period.

**Table 1:** Combined diagnoses proportion

Combined diagnoses	No. of patients	Proportion (%)	Age (years)	Male (%)
Rheumatic heart disease	3	8.8	52	67
Valve disorder	4	11.8	46	75
Hypertension	18	52.9	60	44
Blood hyperviscosity	28	82.4	66	57
Hyperlipidemia	23	67.6	60	39
Myocardial ischemia	11	32.4	67	64
Internal carotid artery insufficiency	4	11.8	58	50
Carotid atherosclerostic plaque	9	26.5	62	56
Diabetes	3	8.8	47	33

### Preoperative characteristics

A total of 34 eyes from 34 patients were studied, including 19 from women (55.9%). The mean age of symptom onset was 44.2 years (SD 2.9, range 22–74 years) (Table 2). The minimum required follow-up period after surgery was 6 months. The mean follow-up period was (9.2 ± 3.3) months (range 6–14 months). Data regarding age, surgical eye, gender, diagnosis, and past history were analyzed. The detailed preoperative demographic data are summarized in Tables 1 and 2 and Figure 1.

**Table 2:** Preoperative patients’ characteristics

Characteristics	Values
**Demographic data**	***n***
Eye (OD/OS)	16/18
Age (years, mean ± SD)	44.2 ± 2.9
Range (years)	22–74
Course of disease (days, mean ± SD)	4 ± 3.6
Range (days)	0.5–9
**Occlusion site**	***n*, (%)**
Hemiretinal artery	3, (8.8)
Inferotemporal	16, (47.1)
Superotempora	11, (32.4)
Under nasal	3, (8.8)
Above nasal	1, (2.9)
**Ophthalmoscopy**	**n, (%)**
Yellow-white emboli	34, (100)
Distal arterial narrowing	34, (100)
Supplied retinal gray and edema	34, (100)
Cotton wool spots	12, (35.3)

Abbreviations: OD, oculus dexter; OS, oculus sinister; SD, standard deviation.

The patients included 3 cases of rheumatic heart disease, 4 cases of cardiac valvular disease, and 18 patients with a history of hypertension. The blood biochemical tests showed 28 patients with high blood viscosity, 23 patients with high blood lipids, 11 patients with acute myocardial ischemia, and 9 patients with carotid atherosclerosis plaque, including 4 patients with carotid atherosclerosis examined with color Doppler ultrasonography. All patients had normal clotting time tests (Figure 1 and Table 1).

Slit lamp biomicroscopy and fundus examination revealed intraretinal edema with inferior retinal whitening in addition to a visible Hollenhorst plaque. FFA revealed an arterial filling defect along the inferotemporal arcade distal to the corresponding embolus with foveal ischemia blocking the zone near the posterior retina in 12 eyes. Cotton wool spots were visible at the occluded retinal site. In the FFA examinations, 34 eyes demonstrated a clearly visible obstruction point, and the dynamic circulation showed obstruction of the blood supply; the distal end of the obstruction and the distal end of the branch arteries were not filled during the early stage, and no fluorescence filling was observed (Figure 2). Retinal blood flow was observed in the distal segment of the retinal artery in 13 eyes. The cilioretinal artery was located in five eyes. The visual field examination indicated the presence of a fixed area shadow or occlusion. The obstruction was present in the inferior retinal artery in 3 eyes, inferior temporal artery in 16 eyes, superior temporal artery in 11 eyes, inferior nasal artery in 3 eyes, and superior nasal artery in 1 eye (Table 2).

### Postoperative characteristics

The emboli were fragmented or moved in all the patients upon administration of the laser pulse. Immediate thickening of the artery diameter was observed, and the retina became slightly red in the blocked area. FFA showed that the blood flow of the artery and its branches was completely restored in 13 eyes (38.2% of patients) (Figure 2d), mostly recovered in 11 eyes (32.4% of patients), and partially restored in five eyes (14.7% of patients); the procedure was ineffective in five eyes (14.7% of patients). Retinal hemorrhages occurred in two eyes, with a mild VH presented in one case. After combined urokinase infusion therapy was administered, the retinal occlusion zone gradually developed a rosy color. The blood flow of the artery was completely restored in 16 eyes (47.1% of patients), mostly recovered in 15 eyes (44.1% of patients), and partially recovered in 3 eyes (8.8% of patients). After the treatment, the retinal artery peak lasted from 3 to 40 s. No retinal vitreous hemorrhaging or systemic complications occurred during the thrombolytic therapy.

### Visual acuity

BCVA was equal to preoperative or improved in 100% (34/34) of patients (Figure 3 and Table 3). Two or more lines of improvement were noted in 19/34 (56%) patients. Four or more lines of improvement were noted for 26% (9/34) patients. Six of the 34 patients (18%) noted six or more lines of improvement. Subgroup analysis was performed to determine outcome after TYE (Table 3). We found no correlation or trend with regard to age, gender, or type of occlusion and visual outcome. Before the procedure, the BCVA ranged from 1.0 to 1.7 in 23 eyes (67.6%), from 0.4 to 0.9 in three eyes (8.8%), from 0.1 to 0.3 in four eyes (8.8%), and was greater than 0 in four eyes (8.8%). At the most recent visit, conscious visual improvement after treatment was discussed with the patients, and the BCVA ranged from 1.0 to 1.7 in nine eyes (26.5%), from 0.4 to 0.9 in 12 eyes (35.3%), from 0.1 to 0.3 in seven eyes (20.6%), and was greater than 0 in six eyes (17.6%) (Figures 3 and 4). The difference in the visual acuity between pre- and post-treatment measurements was statistically significant (*χ*^2^ = 30.7, *P*<0.05). Perimetry showed that fading of the original regional shadow varied in 32 eyes, and no change occurred in two eyes.

**Table 3:** Subgroup Analysis of TYE combined with urokinase thrombolysis

Sub group	TYE				TYE combined with urokinase thrombolysis			
	No. of patients	Age (years)	Male (%)	Duration (d)	No. of patients	Age (years)	Male (%)	Duration (d)
0-1 line	25	52	48	10	15	48	53	5
>2 lines	9	48	67	6	19	52	42	10
>4 lines	5	50	60	12	9	56	77	9
>6 lines	3	56	67	9	6	46	67	7

Abbreviation: TYE, transluminal Nd:YAG laser embolysis.

## Discussion

BRAO might be a result of platelet-fibrin emboli, cholesterol plaques, or calcified emboli that typically lodge at the bifurcation of retinal arterioles or at a narrowing of a vessel. Obstruction of the blood flow results in partial or complete retinal ischemia and sudden loss of vision [6]. Numerous treatment modalities have been attempted for hemi and retinal artery branch occlusion without notable success. Low-intensity photocoagulation with an argon laser has been used to treat cholesterol emboli associated with BRAO, which resulted in prompt reperfusion of the retina [3–5]. No immediate treatment effect was observed. The use of thrombolysis for the treatment of BRAO has been the subject of intense investigation. Most authors have attempted to address this issue with intra-arterial thrombolysis [7]. The available data on intravenous thrombolysis are largely limited to isolated case reports or retrospective case series.

The authors employed an Nd:YAG laser to photo disrupt the emboli and administered fibrinolysis therapy via peripheral intravenous infusion of urokinase within the hemi or branch retinal arteries to achieve reperfusion of the retina.

The Nd:YAG laser has a wavelength of 1064 nm, uses infrared light, and has a precise laser focus spot with small, penetrating characteristics. An iridectomy can be applied to the vitreous bands before macular retinal hemorrhage drainage occurs, except in the case of cataracts, intraocular lens front film, and posterior synechia [8–10]. The retinal artery wall absorbs less infrared light; therefore, low energy radio frequencies should be used for an arterial embolus due to the ionizing effect, which results in cutting of the tissue and the formation of plasma in the target tissue. The shock wave produced by the tissue can disrupt the emboli without harming the arterial wall. The use of Nd:YAG laser treatment for BRAO has rarely been reported. We used Nd:YAG laser pulses to conduct thrombectomies in this group of hemi and branch RVO patients with retinal artery emboli that had been disrupted or translocated, and the blocked artery diameter was immediately enlarged. The treated artery trunk and its branches exhibited completely restored blood flow in 38.2% of the cases, basic recovery in 32.4% of the cases, and partial recovery in 14.7% of the cases; the treatment was ineffective in five eyes (14.7%). A retinal hemorrhage occurred in two eyes following the laser treatment, which presented as a mild VH. Nd:YAG laser treatment using pulses for hemi and branch RVO has certain advantages and a high safety rate. The two cases of retinal hemorrhage resulted from poor patient cooperation. Minor position changes during the laser pulses could cause bias without damage to the retinal blood vessels in patients exhibiting good cooperation. Some reports state that the use of argon ion laser pulses is therapeutically effective for the treatment of arterial emboli, but the wavelength of the laser causes heat damage and is easily absorbed by hemoglobin, resulting in vascular endothelial damage and thrombosis. The Nd:YAG laser thrombectomy does not produce a thermal effect and therefore does not cause these complications.

Urokinase catalyzes the cleavage of plasminogen to plasmin and could degrade a fibrin clot by thrombolysis and produce rapid and positive results. It has short half-life of approximately 16 min, improves vascular adenosine diphosphate (ADP) activity, inhibits ADP-induced platelet aggregation, and prevents thrombosis [11,12]. In this group of patients, Nd:YAG laser thrombolytic therapy combined with surgery and FFA improved the blood flow more than simple laser surgery and significantly improved the blocked artery, resulting in no bleeding, concurrent abnormalities, or other systemic diseases. The treated artery trunk and its branches exhibited completely restored blood flow in 47.1% of the cases, basic recovery in 44.1% of the cases, and partial recovery in 8.8% of the cases, indicating that patients who underwent Nd:YAG laser treatment combined with thrombolytic therapy exhibited further dissolution of small emboli with an improved therapeutic effect.

Mason et al. [13] reported that the vision prognosis after BRAO appeared to correlate with the presenting visual acuity (VA) and that eyes with an initial VA of 20/40 or better typically remained at 20/40 or better. Individuals with poor VA (e.g. 20/100 or worse) generally did not show significant improvement. The researchers concluded that it might be misleading to indicate that 80% of eyes with BRAO improve to 20/40 or better (as described in previous reports) when the final BCVA is so closely related to the presenting BCVA. Damage to the hemi and macular branch of during retinal artery occlusion, regardless of whether it is dependent on cilia or the macular retinal artery, is closely related to the quality of the central vision. The retinal artery occlusion site was different in this group of patients, and thus the effects on vision were significantly different. For these patients, we could not simply evaluate the efficacy of the central vision. FFA could be used to determine retinal vein circulation, the range of obstructive vascular injury, and whether the macular circulation was impaired, which demonstrated its importance for the effectiveness of disease treatment. Retinal artery occlusion after treatment and before the peak fluorescence duration of 3–40 s might explain the persistence and extent of the obstruction severity, and a shorter time allowed retinal circulation to recover. In this group of patients, at 12 h to 9 d after the onset of treatment, most of the patients had missed the opportunity for the best timing of treatment. FFA showed partial recovery in three patients but produced no improvement in vision. The blocked artery supply area showed completely irreversible retinal damage.

Recent reports have shown immediate restoration of retinal blood flow after TYE, in addition to rapid restoration of the BCVA [14,15]. Complications such as vitreous subretinal hemorrhages were observed. In these cases, laser treatment resulted in dramatic improvement in the BCVA and restoration of the retinal blood flow despite a small amount of vitreous hemorrhaging.

Rapid reperfusion of the retina is associated with anatomical and visual acuity improvement. TYE combined with intravenous urokinase thrombolysis should be considered an effective treatment for patients with BRAO with visible emboli. The present study is a pilot study that was undertaken to examine the treatment of BRAO, and the absence of a control group is a limitation of the present study. In the future, more cases will be included, according to the preliminary data obtained in the present study.

## Clinical perspectives

Branch retinal artery occlusion (BRAO) result in partial or complete retinal ischemia and sudden loss of vision. In most instances, these conditions are thought to be caused by an embolus, intraluminal thrombus, or atherosclerotic plaque. These conditions most commonly occur in older adults with a history of systemic hypertension, diabetes mellitus, carotid atherosclerosis, or cardiac valvular disease. Our study certified TYE combined with urokinase thrombolysis is effective for reperfusion of occluded branch retinal arteries and improving visual recovery in patients with visible emboli. Rapid reperfusion of the retina is associated with anatomical and visual acuity improvement. TYE combined with intravenous urokinase thrombolysis should be considered an effective treatment for patients with BRAO with visible emboli. This study is a pilot study that was undertaken to examine the treatment of BRAO.

## Abbreviations

BCVA: best-corrected visual acuity
BRAO: branch retinal artery occlusion
CRAO: central retinal artery occlusion
FFA: fundus fluorescein angiography
OCT: optical coherence tomography
TYE: transluminal Nd:YAG laser embolysis
